# Supplementary figures and images for: Echis carinatus snake venom metalloprotease-induced toxicities in mice: Therapeutic intervention by a repurposed drug, Tetraethyl thiuram disulfide (Disulfiram)
Source: PLoS Negl Trop Dis. 2021 Feb 2;15(2):e0008596. doi: 10.1371/journal.pntd.0008596 (PMC7880489; doi:10.1371/journal.pntd.0008596)

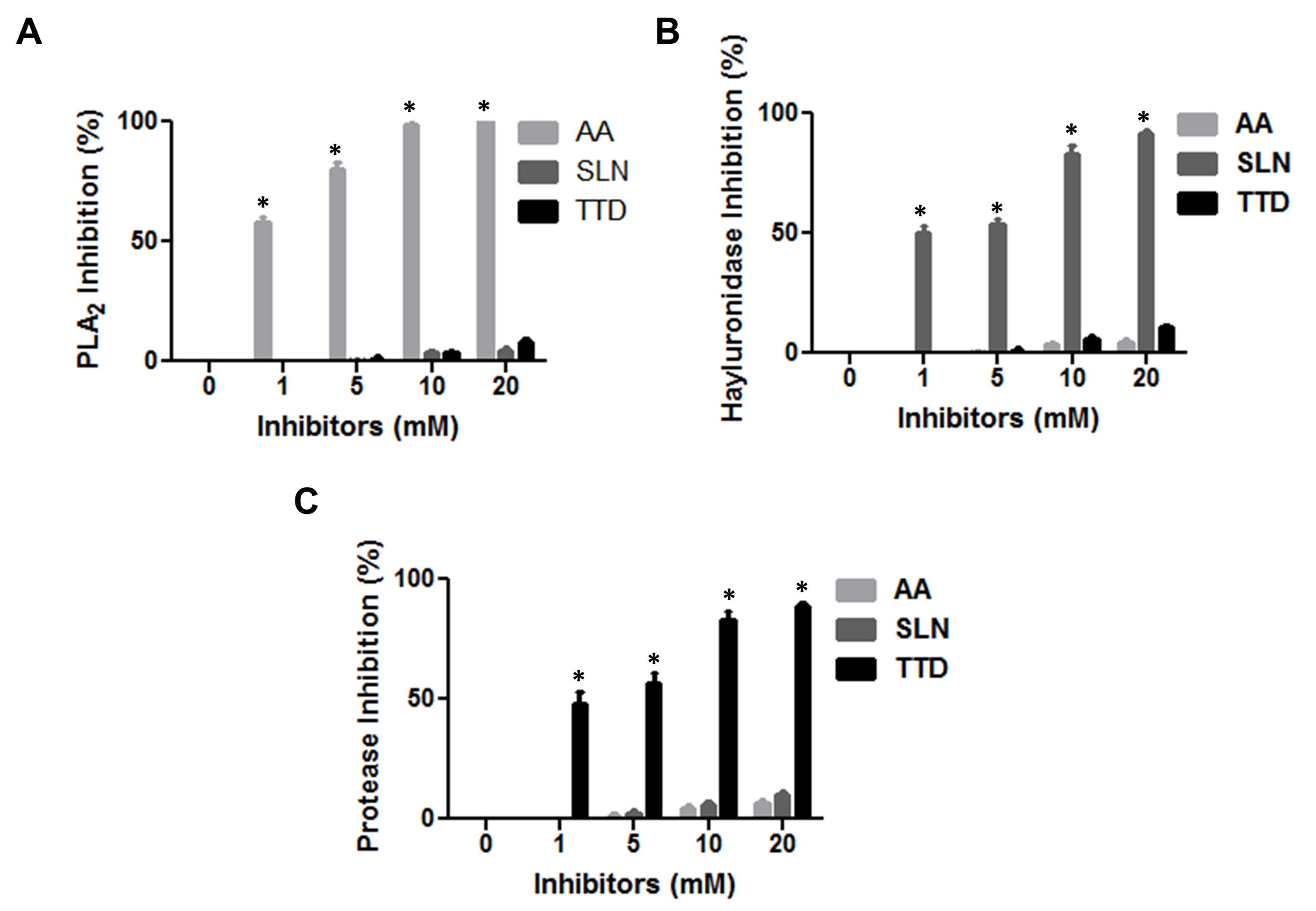

Supplement: S1 Fig — ECV was pre-incubated without or with various concentrations of AA/TTD/SLN at 37°C for 5 min and subjected for PLA2 (A), hyaluronidase (B) and protease (C) activity. The inhibition was represented as % inhibition and venom alone considered as 100% activity. * p < 0.05, when compared ECV versus ECV + AA, ECV + SLN and ECV + TTD. (TIF) [file pntd.0008596.s001.tif]

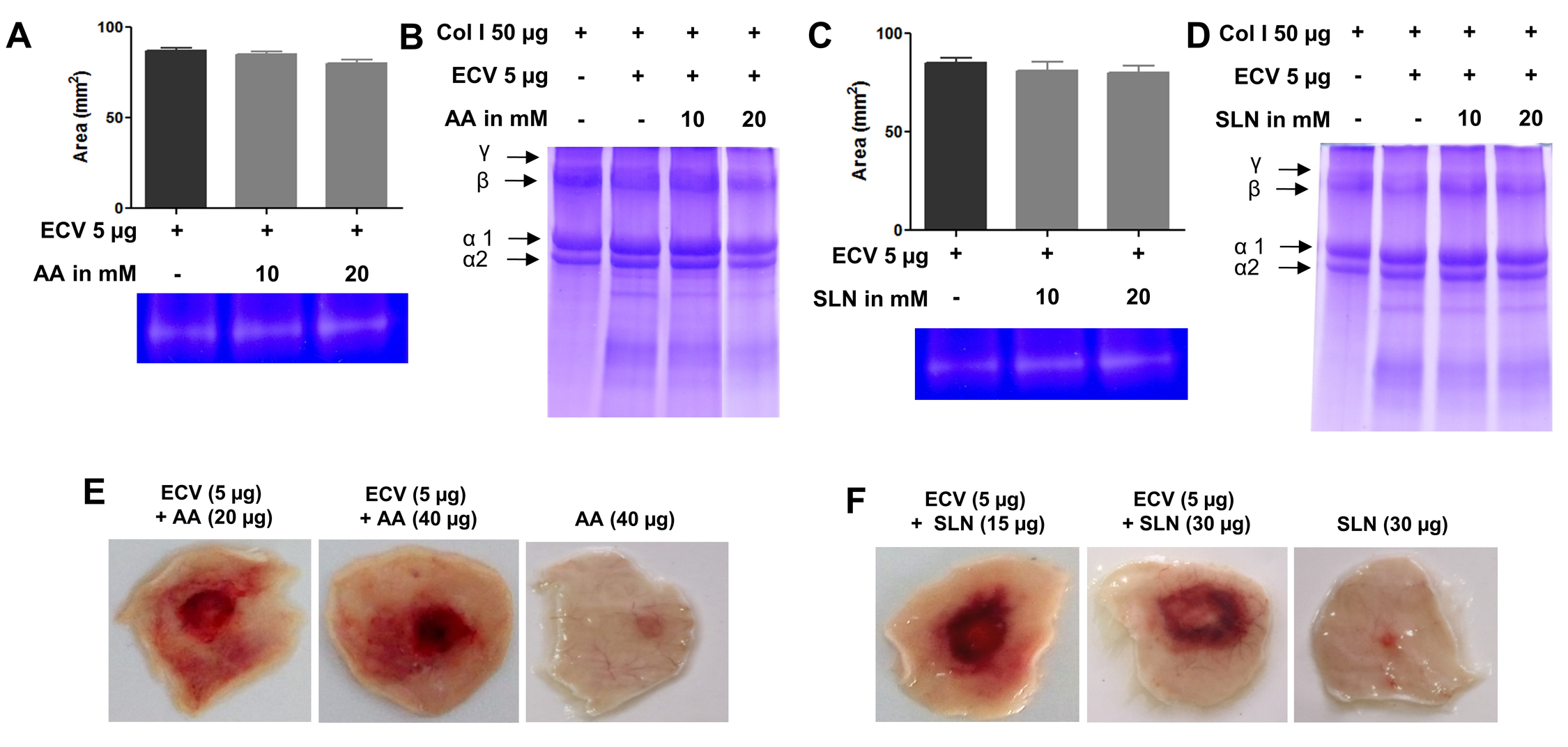

Supplement: S2 Fig — ECV was pre-incubated without or with different concentrations of either AA (A) or SLN (C) at 37°C for 5 min and subjected to gelatin zymogram as described in methods section. Clear zones in the gel indicate the hydrolysis of gelatin by ECV. Area of gelatinolytic activity was measured using graph sheet represented as area (mm2) (A and C). For collagen I (Col I), degradation, ECV was pre-incubated without or with increased concentrations of either AA (B) or SLN (D). Pre-incubated reaction mixture of ECV and inhibitors were further incubated with 50 μg of collagen I for 3 h at 37°C and cleavage pattern was analyzed using 7.5% SDS-PAGE and visualized by staining with CBB-G250. For skin hemorrhage, mice were injected (n = 3; i.d.) with 5 μg of ECV followed by two different concentrations of AA and SLN after 30 min venom injection. After 180 min, dorsal patches of mice skin were photographed (E and F). Data are representative of two independent experiments. (TIF) [file pntd.0008596.s002.tif]

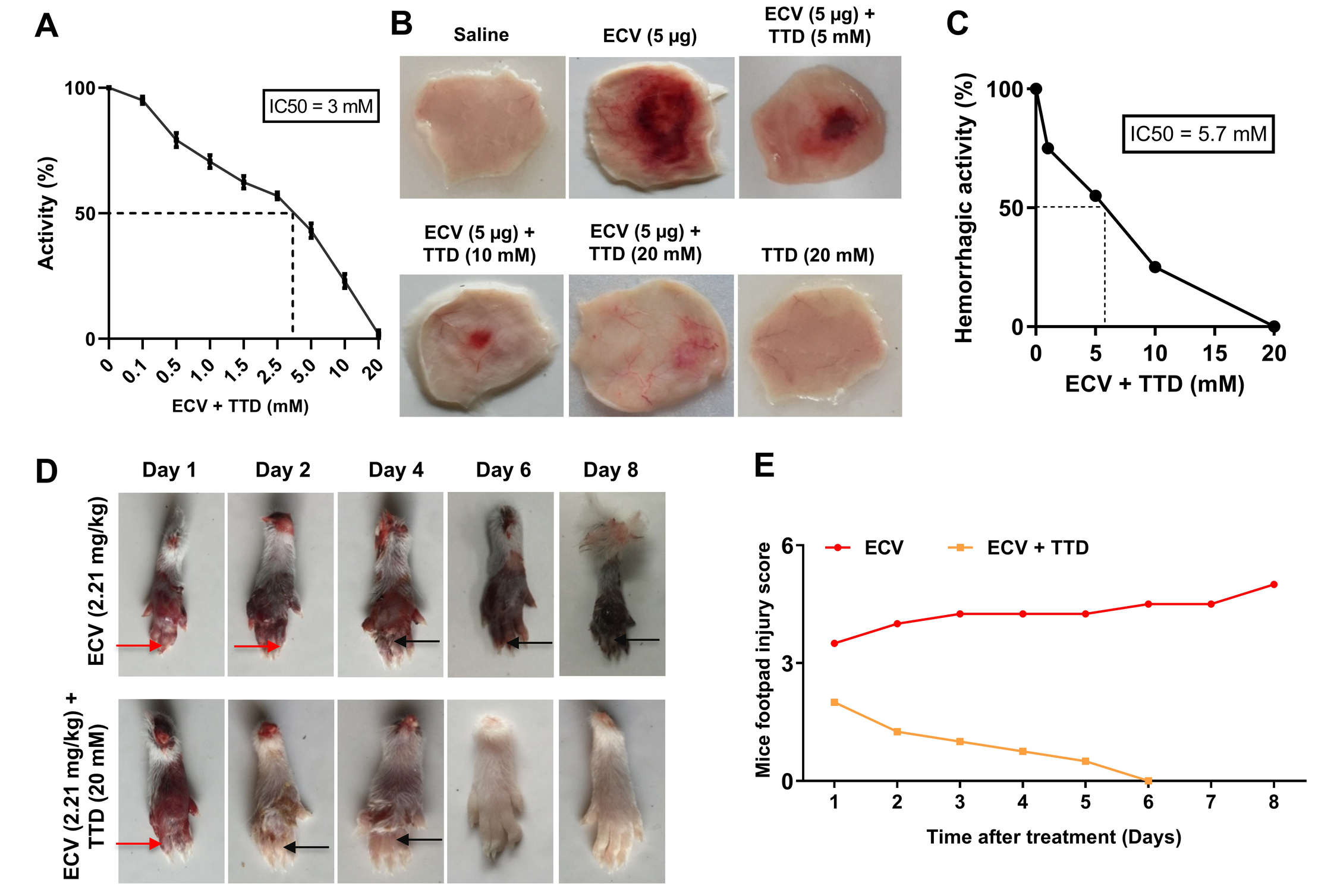

Supplement: S3 Fig — Reaction mixture (1 ml) contained 0.4 ml of casein (2%) in 0.2 M Tris-HCl buffer pH 8.5 was incubated for 150 min at 37°C with 25 μg of ECV and various concentrations of TTD (0–20 mM). The inhibition was represented as % inhibition and IC50 (median inhibitory concentration) of the TTD was calculated (A). For inhibition of skin hemorrhage, mice were injected (i.d.) with 5 μg of ECV that was pre-incubated with different concentrations of TTD (0–20 mM) at 37°C for 5 min. After 180 min, dorsal patches of mice skin were photographed and IC50 (median inhibitory concentration) of the TTD was calculated (B and C). For inhibition of tissue necrosis, mice footpads were injected with ECV (LD50; 2.21 mg/kg) pre-incubated with TTD (20 mM) at 37°C for 5 min and footpads were photographed from day 1 to day 8 (D). Red arrow indicates edema and black arrow indicates tissue necrosis. ECV-induced footpad injury was measured manually on a scale of 1 to 5 (E). Data are representative of two independent experiments. (TIF) [file pntd.0008596.s003.tif]

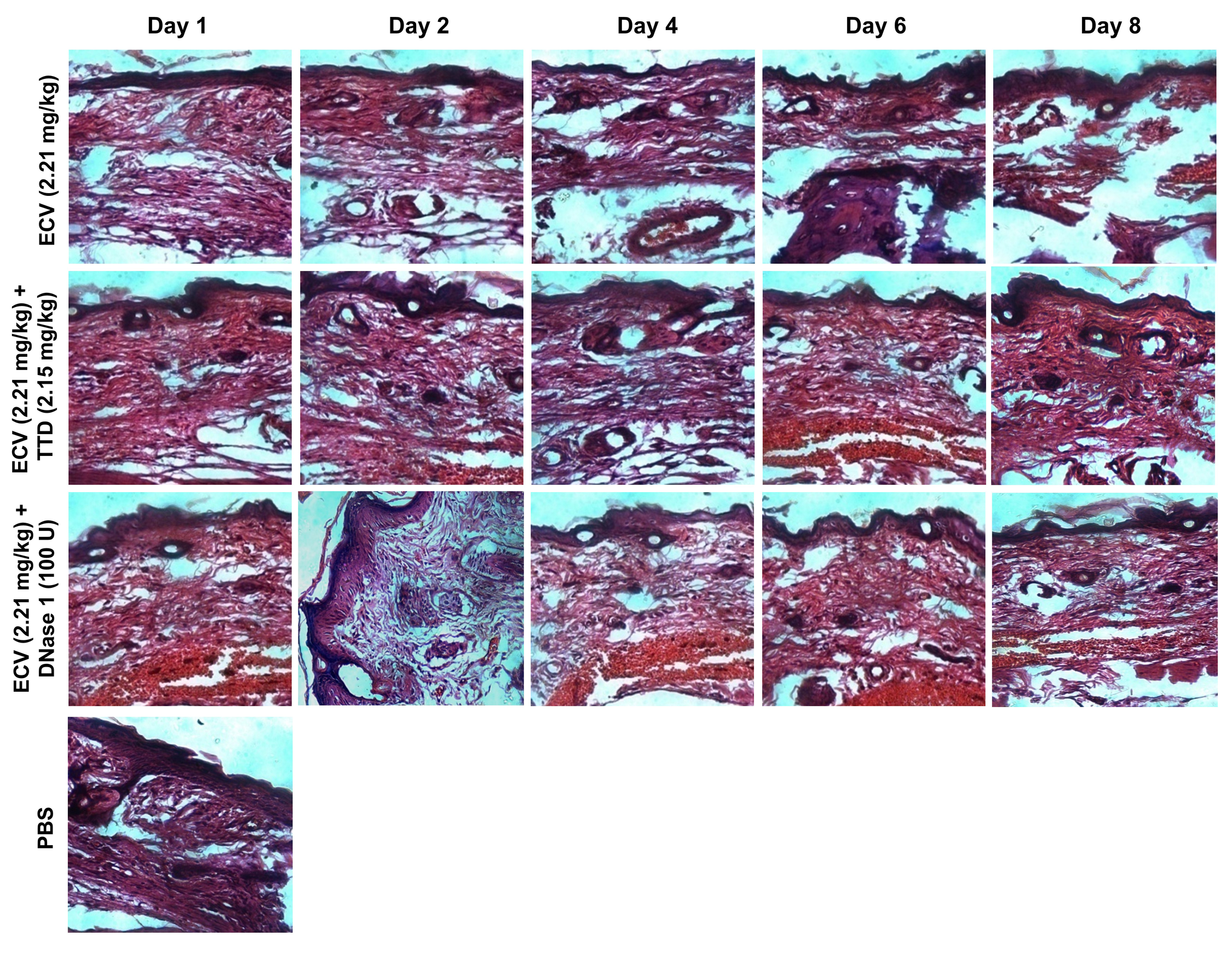

Supplement: S4 Fig — Mice footpad was injected with ECV followed by TTD or DNase 1 injection (30 min post venom injection). Mice were euthanized and footpad tissues were processed for histological sections and analyzed for tissue damage by H & E staining. PBS injected mouse footpad serves as control. (TIF) [file pntd.0008596.s004.tif]

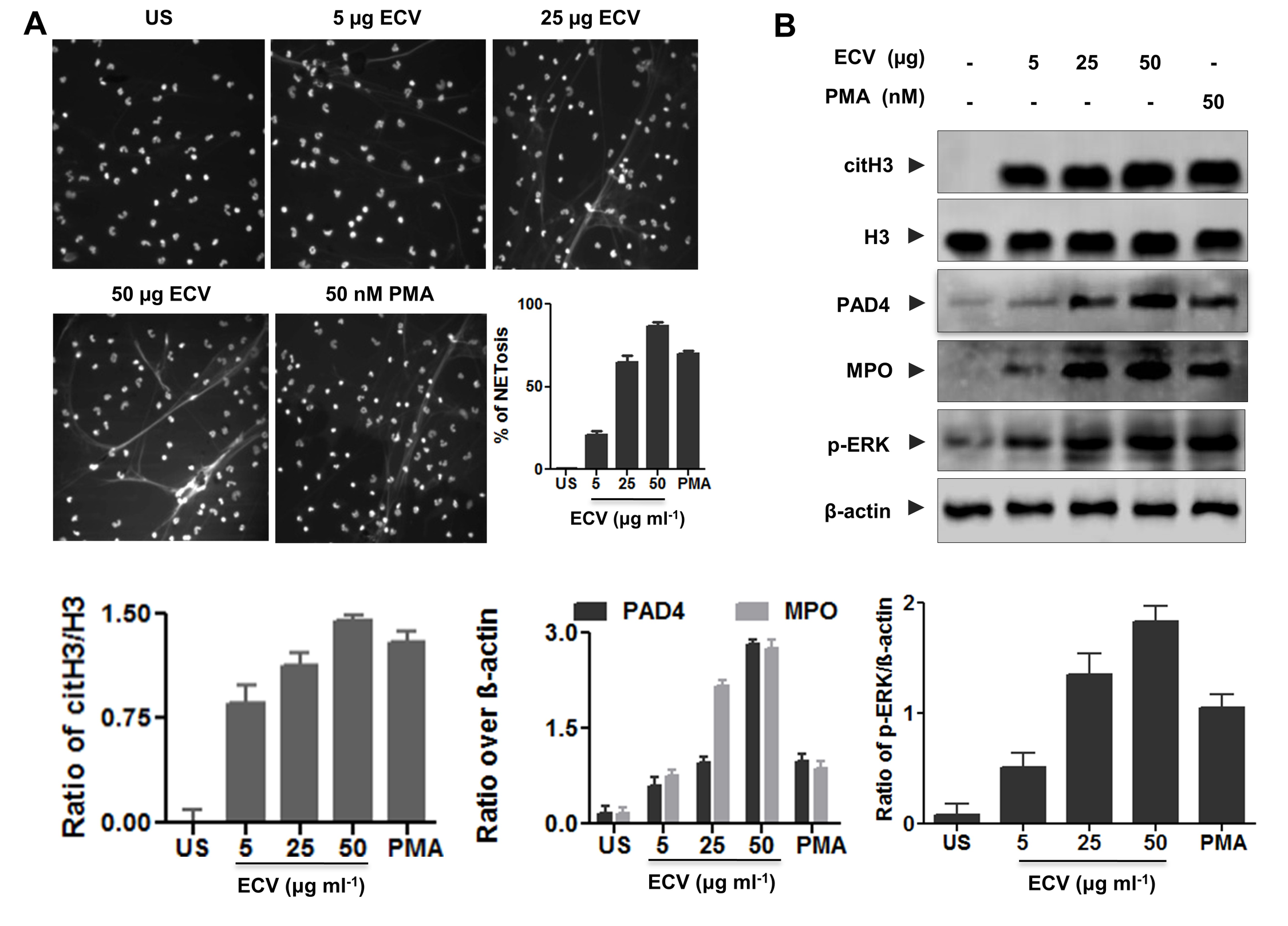

Supplement: S5 Fig — Human neutrophils were stimulated with ECV for 180 min and NETs formation was observed and quantitated (A). The whole cell lysates were analyzed for the phosphorylated ERK and NETosis markers using Western blotting. The p-ERK, MPO and PAD4 were quantitated using β-actin as a loading control and H3 as loading control for citH3. Data are representative of two independent experiments. (TIF) [file pntd.0008596.s005.tif]

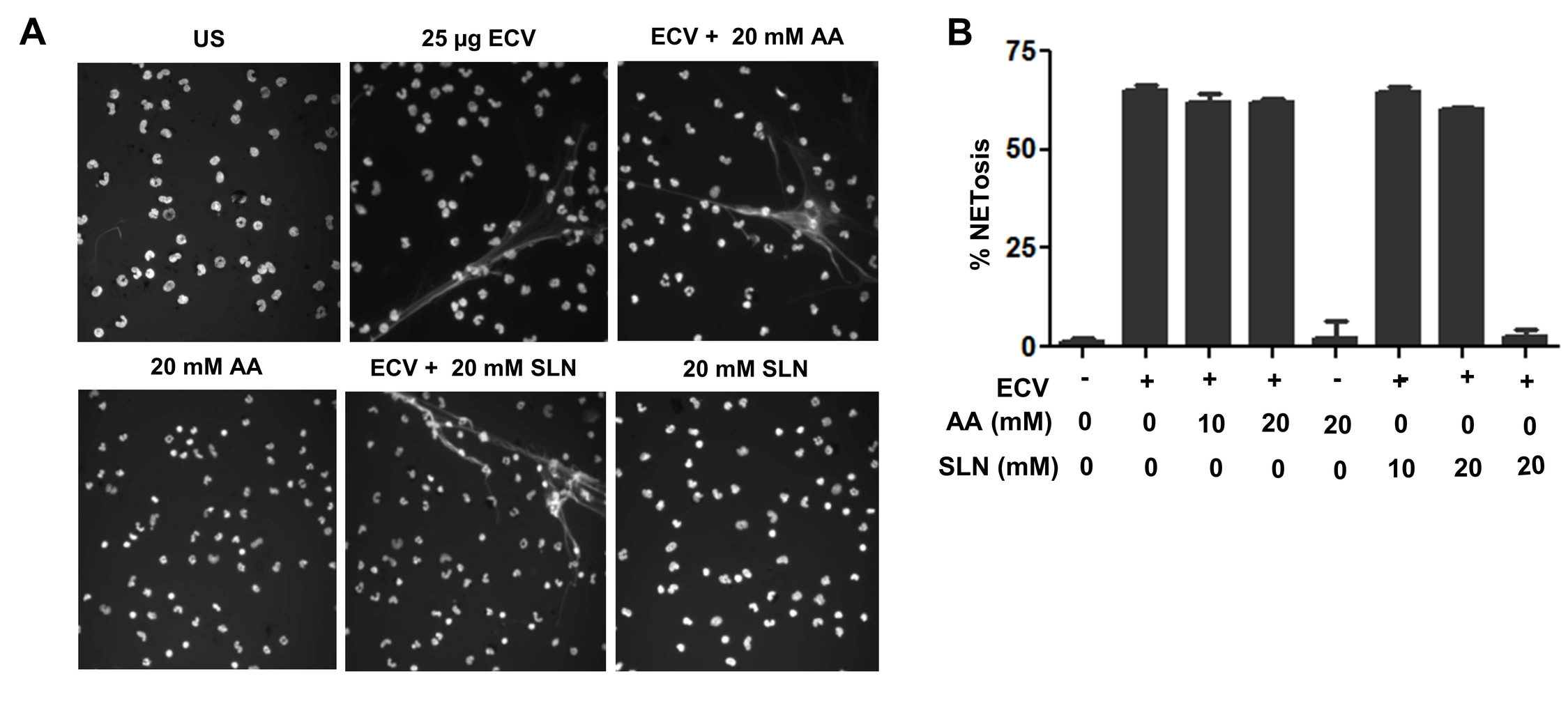

Supplement: S6 Fig — Human neutrophils were stimulated (180 min) with ECV pre-incubated with different concentrations of either AA or SLN for 5 min at 37°C and NETs formation was photographed under microscope (A) and quantitated as percent NETosis (B). The data represent the mean ± SD of three independent experiments. * p < 0.05, when compared ECV versus ECV + AA and ECV + SLN. (TIF) [file pntd.0008596.s006.tif]
