# Supplementary material for: Echis carinatus snake venom metalloprotease-induced toxicities in mice: Therapeutic intervention by a repurposed drug, Tetraethyl thiuram disulfide (Disulfiram)
Source: PLoS Negl Trop Dis. 2021 Feb 2;15(2):e0008596. doi: 10.1371/journal.pntd.0008596 (PMC7880489; doi:10.1371/journal.pntd.0008596)
Supplement: Attachment — Submitted filename: Response to reviewers comments PNTD.docx [file pntd.0008596.s007.docx]

Authors would like to thank the editor for considering our manuscript for revision in esteemed journal, **PLOS Neglected Tropical Diseases**. We also thank all the reviewers for critically evaluating the manuscript. The comments raised by reviewers are scientifically valid and we have addressed the comments as detailed below. We have provided additional experimental details and more evidence to support the manuscript. We have also included some new data in the form of supplementary information.

**Methods**

-Are the objectives of the study clearly articulated with a clear testable hypothesis stated?

-Is the study design appropriate to address the stated objectives?

-Is the population clearly described and appropriate for the hypothesis being tested?

-Is the sample size sufficient to ensure adequate power to address the hypothesis being tested?

-Were correct statistical analysis used to support conclusions?

-Are there concerns about ethical or regulatory requirements being met?

**Reviewer #1**

**Comment**

The two methods most likely to result in questions refer to other papers and "modified appropriately". Since these involve quite graphic images, I think it is better to be explicit about how this was done and most importantly, how the animals were kept comfortable. For example, the animals were sedated for the Disulfiram paw injection, but what about the venom injection? for the intradermal and paw injection this is my advice, but I would leave it to the authors to decide how best to handle.

**Response:** Mice were kept in separate cages at humidity and temperature controlled room and had free access to the standard laboratory diet and water. During all experiments, animal care and handling were in accordance with the guidelines of the Committee for the Purpose of Control and Supervision of Experiments on Animals (CPCSEA). During animal experiments mice were anesthetized with pentobarbitone (30 mg/kg; i.p.) and then venom/Disulfiram injection was done.

**Comment**

"The signs and symptoms of toxicities were observed up to 24 h and survival time was recorded. For systemic hemorrhage and bleeding studies, mice received ECV (LT50; 2.21 mg/kg; i.p.) and challenged with TTD (2.15 mg/kg; i.p.) or ED ASV, 30 min post ECV injection. After 3 h peritoneal cavity was observed for symptoms and photographed."

Here I think you have substituted "symptoms" where you mean "signs". Symptoms are "headache, back pain, abdominal pain" (subjective) whereas signs are things like, "hemorrhage, weakness, guarding" (physically observable).

**Response:** From the reviewers comment we clearly understood the difference between signs and symptoms. We agree with the reviewer that signs and symptoms are unlike abnormalities that can indicate a potential medical condition. In this manuscript we have observed only sign of the peritoneal cavity of the venom injected mice. As per the reviewer suggestions, the sentence “The signs and symptoms of toxicities were observed up to 24 h and survival time was recorded” is changed to “The signs of toxicities were observed up to 24 h and survival time was recorded” and incorporated in the revised manuscript.

**Reviewer #2**

-Are the objectives of the study clearly articulated with a clear testable hypothesis stated? Generally yes

-Is the study design appropriate to address the stated objectives? Partially, see comments below.

-Is the population clearly described and appropriate for the hypothesis being tested? Generally yes.

-Is the sample size sufficient to ensure adequate power to address the hypothesis being tested? Yes

-Were correct statistical analysis used to support conclusions? Partially, see comments below

-Are there concerns about ethical or regulatory requirements being met? No concerns

**Comment**

The use of inhibitors of PLA_2_ and hyaluronidase activities is interesting and relevant for assessing the possible role of these other venom enzymes in the pathogenesis of tissue damage. However, the authors need to show (controls) that such inhibitors, at the concentrations used, indeed inhibited enzymatic PLA_2_ and hyaluronidase activities. These controls are necessary.

**Response:** In the following experiment, we have used the reported inhibitors for PLA_2_ and hyaluronidase enzymes. Many reports suggested that Aristolochic acid and Silymarin can inhibit the snake venom PLA_2_ and hyaluronidases, respectively in a concentration dependent manner.

As reviewer suggested, we are providing additional experimental details of inhibition of three major enzymes of the snake venom using specific inhibitors to support the manuscript. We have included these new data as **Fig. S1**.

**Fig. S1:** **Inhibition of ECV-induced enzymatic activities by specific inhibitors.** ECV was pre-incubated without or with various concentrations of AA/TTD/SLN at 37ºC for 5 min and subjected for PLA_2_ **(A)**, hyaluronidase **(B)** and protease **(C)** activity. The inhibition was represented as % inhibition and venom alone considered as 100% activity. * p < 0.05, when compared ECV versus ECV + AA, ECV + SLN and ECV + TTD.

**Comment**

ECV-induced hemorrhage, line 5: Use ‘various doses of…’ instead of ‘various concentrations’

**Response:** As per reviewer suggestions, we have revised the sentence to ‘various doses of’----instead of ‘various concentrations’

**Comment**

Description of the necrosis model in footpad: The semi quantitative scale used for grading the extent of tissue damage includes the ‘necrosis’. From the text it seems that such effect was judged visually, i.e., macroscopically. It is difficult to identify necrosis without doing histological analysis. Was this done to corroborate whether the macroscopic observations agree with histological assessment? It is recommended that the macroscopic observations of necrosis be complemented by histological observations, at least with the crude venom alone.

**Response:** We are providing experimental details, histological analysis to support the manuscript. We have also included the histological assessment data in the form of supplementary information **(Fig. S4)**.

**Fig. S4: Histochemical staining of ECV-induced tissue necrosis in mice footpad and its inhibition by TTD.** Mice footpad was injected with ECV followed by TTD or DNase 1 injection (30 min post venom injection). Mice were euthanized and footpad tissues were processed for histological sections and analyzed for tissue damage by H & E staining. PBS injected mouse footpad serves as control.

**Comment**

The authors need to briefly explain why DNase was used as positive control in this assay.

**Response:** Katkar et al. showed that the ECV-induced NETosis-venom complex is responsible for the continued tissue destruction at the bitten site. Further, DNase administration accelerates the healing of chronic wounds by degrading the deposited NETs”. Hence, DNase 1 was used as a positive control for the neutralization of ECV-induced NETosis responsible for tissue necrosis.

**Comment**

In the necrosis assay, the inhibition experiments described include a protocol in which the inhibitor was administered after venom injection. Did the authors also carried out experiments with pre-incubation of venom and inhibitors? The same holds for the inhibition of hemorrhagic activity. Were pre-incubation experiments also done? Pre-incubation type experiments are necessary to determine the inhibitor IC50 (median inhibitory concentration), which enables the comparison with other SVMP inhibitors previously published. It is recommended that the authors do these pre-incubation inhibition experiments, and determine the IC50.

**Response:** We have performed experiments with pre-incubation of ECV with inhibitors and determined IC_50_ for caseinolytic activity, hemorrhagic activity and footpad necrosis assay. The data have been included as **Fig. S3A - S3E** in the revised manuscript.

**Fig. S3: Inhibition of ECV-induced protease activity and local toxicities by TTD.** Reaction mixture (1 ml) contained 0.4 ml of casein (2%) in 0.2 M Tris-HCl buffer pH 8.5 was incubated for 150 min at 37ºC with 25 μg of ECV and various concentrations (0 - 20 mM) of TTD. The inhibition was represented as % inhibition and IC_50_ (median inhibitory concentration) of the TTD was calculated **(A)**. For inhibition of skin hemorrhage, mice were injected (i.d.) with 5 µg of ECV that was pre-incubated with different concentrations of TTD at 37ºC for 5 min. After 180 min, dorsal patches of mice skin were photographed and IC_50_ (median inhibitory concentration) of the TTD was calculated **(B and C)**. For inhibition of tissue necrosis, mice footpads were injected with ECV (LD50; 2.21 mg/kg) pre-incubated with TTD (2.15 mg/kg) at 37ºC for 5 min and footpads were photographed from day 1 to day 8 **(D)**. Red arrow indicates edema and black arrow indicates tissue necrosis. ECV-induced footpad injury was measured manually on a scale of 1 to 5 **(E)**. Data are representative of two independent experiments.

**Comment**

Experiments on lethality, third line: LD50 instead of LT50. In the lethality experiments the authors used the pre-incubation protocol in addition to the ‘rescue’ protocol. Why was this not done with hemorrhage and footpad necrosis?

**Response:** According to reviewer suggestions, we have replaced the LT_50_ by LD_50_.

For the lethality/survival time analysis experiments, we have initially screened the anti-venom potentials of TTD in pre-incubation experiments. Upon confirmation, we have conducted the challenging experiments (Post injection).

In case of local tissue necrosis, the experiments were performed with pre-incubation of venom and inhibitors for both hemorrhagic activity and footpad necrosis. Upon confirmation with pre-incubation experiments, we have performed the challenging experiments (Post injection). As per the reviewer suggestions, we have also included the pre-incubation experimental data as **Fig. S3A - S3E**.

**Fig. S3: Inhibition of ECV-induced protease activity and local toxicities by TTD.** Reaction mixture (1 ml) contained 0.4 ml of casein (2%) in 0.2 M Tris-HCl buffer pH 8.5 was incubated for 150 min at 37ºC with 25 μg of ECV and various concentrations (0 - 20 mM) of TTD. The inhibition was represented as % inhibition and IC_50_ (median inhibitory concentration) of the TTD was calculated **(A)**. For inhibition of skin hemorrhage, mice were injected (i.d.) with 5 µg of ECV that was pre-incubated with different concentrations of TTD at 37ºC for 5 min. After 180 min, dorsal patches of mice skin were photographed and IC_50_ (median inhibitory concentration) of the TTD was calculated **(B and C)**. For inhibition of tissue necrosis, mice footpads were injected with ECV (LD50; 2.21 mg/kg) pre-incubated with TTD (2.15 mg/kg) at 37ºC for 5 min and footpads were photographed from day 1 to day 8 **(D)**. Red arrow indicates edema and black arrow indicates tissue necrosis. ECV-induced footpad injury was measured manually on a scale of 1 to 5 **(E)**. Data are representative of two independent experiments.

**Comment**

**Statistics:** A non-parametric test (Mann-Whitney U) was used to compare pairs of means, whereas a parametric test (ANOVA) was used for comparing means of more than two groups. If the authors decided that non-parametric tests were to be used, either because there is no evidence of a normal distribution of data or because the n is low, then both types of analyses should use non parametric tests and, therefore, Kruskal-Wallis test has to be used instead of ANOVA when comparing more than two groups.

**Response:** We have used one way ANOVA to compare more than two groups. We have modified Statistics section in the revised manuscript accordingly.

**Reviewer #3**

(No Response)

**Results**

-Does the analysis presented match the analysis plan?

-Are the results clearly and completely presented?

-Are the figures (Tables, Images) of sufficient quality for clarity?

**Reviewer #1**

**Comment**

I have no question about the results. This is a very thorough investigation. It would be useful to have some clinical vision to illustrate how the authors might see this used and how the experimental doses compare to clinical observations.

For example, in my experience, Echis bite patients don't die immediately. The inflammatory syndrome appears over days. Does this mean high dose IV in the hospital is a viable concept or should it be an oral field treatment?

The authors admirably used rapidly lethal doses (hours). This speaks well to the concept. They should emphasize this. I doubt few people are envenomed with 3mg/kg venom. How would this drug be anticipated to perform at a lower dose, but more common bite presentation (for discussion eg). Would the dose of drug be lower and more plausible for pocket carriage?

**Response:** Although our study is focused on preclinical setup it is interesting to extrapolate to clinical setup. Since TTD is an Antabuse drug and it is given orally, we feel that it will be an oral field treatment as first aid therapy.

As reviewer doubted, it is very hard to predict people are envenomed with 3 mg/kg venom. And also, without clinical studies it is hard to discuss about the dose of drug. However, TTD exerts its effect in dose dependent manner and we believe that the dose of drug will be lower if venom concentration is low in envenomed people. Since snakebite is a medical emergency it is better to administer the dose which is accepted in the treatment of alcohol abuse therapy.

**Reviewer #2**

-Does the analysis presented match the analysis plan? Yes

-Are the results clearly and completely presented? Generally yes.

-Are the figures (Tables, Images) of sufficient quality for clarity? Yes.

**Comment**

Inhibition of gelatin hydrolysis by venom: As shown in Fig 1, not even a concentration of 20 mM of the inhibitor inhibited the activity. In the text, the authors claim ‘maximal inhibition was observed at 20 mM TTD”. The sentence is a bit confusing, because no complete inhibition was achieved and also because it is likely that higher inhibitor concentration would have a higher inhibitory activity. The inhibition of proteolytic activity of venom on various ECM substrates is more clear and well shown.

**Response:** According to the reviewer suggestion, we have modified “maximal inhibition was observed at 20 mM TTD” to “TTD inhibited gelatinolytic activity in concentration dependent manner”

**Comment**

Inhibition of hemorrhage, as noted above, it is recommended that the authors carry out experiments with pre-incubation of venom and various concentration of the inhibitor in order to determine the IC_50_.

**Response:** According to the reviewer suggestion, we have performed pre-incubation experiments for inhibition of hemorrhagic activity and IC_50_ (median inhibitory concentration) of the TTD was determined (12 μg) and included in **Fig. S3B and S3C**.

**Fig. S3: Inhibition of ECV-induced protease activity and local toxicities by TTD.** Reaction mixture (1 ml) contained 0.4 ml of casein (2%) in 0.2 M Tris-HCl buffer pH 8.5 was incubated for 150 min at 37ºC with 25 μg of ECV and various concentrations (0 - 20 mM) of TTD. The inhibition was represented as % inhibition and IC_50_ (median inhibitory concentration) of the TTD was calculated **(A)**. For inhibition of skin hemorrhage, mice were injected (i.d.) with 5 µg of ECV that was pre-incubated with different concentrations of TTD at 37ºC for 5 min. After 180 min, dorsal patches of mice skin were photographed and IC_50_ (median inhibitory concentration) of the TTD was calculated **(B and C)**. For inhibition of tissue necrosis, mice footpads were injected with ECV (LD50; 2.21 mg/kg) pre-incubated with TTD (2.15 mg/kg) at 37ºC for 5 min and footpads were photographed from day 1 to day 8 **(D)**. Red arrow indicates edema and black arrow indicates tissue necrosis. ECV-induced footpad injury was measured manually on a scale of 1 to 5 **(E)**. Data are representative of two independent experiments.

**Reviewer #3**

**Comment**

(No Response)

**Conclusions**

-Are the conclusions supported by the data presented?

-Are the limitations of analysis clearly described?

-Do the authors discuss how these data can be helpful to advance our understanding of the topic under study?

-Is public health relevance addressed?

**Reviewer #1**

As above, I think two areas of discussion are lacking and the authors might be short-changing themselves

**Comment**

What is the most common natural history presentation of Echis bite (rapid lethality or inflammatory/coagulopathy syndrome over period of days)? This puts clinical relevance and natural history into context and is very educational for readers, too.

**Response:** This is a very interesting to consider the most common natural history presentation of *Echis* bite. As reviewer suggestion, we have included the following information in the discussion section.

There are several case reports that reported the major complications related to hemotoxic snakebite including *Echis carinatus*. *Echis carinatus* bite induces hematological complications, local pain, bleeding and edema at the bite site. Untreated *Echis* envenomation may involve multiple organs and the patient may suffer from, hematuria, renal failure, hemorrhage, anemia, hypotension and disseminated intravascular coagulation with capillary leak syndrome. The LD_50_ of *Echis carinatus* envenomation is 6.65 mg/kg and an average bite may yield about 40 mg of venom (Clinical Toxinology in Asia Pacific and Africa pp 209-232; J. Venom. Anim. Toxins incl. Trop. Dis vol.12 no.1 Botucatu 2006; Am. J. Trop. Med. Hyg., 86(2), 2012, pp. 254–257).

Generally, envenomation by EC is associated with a mortality rate of 10 - 20% if there is no initiation of effective treatment early. The major cause of mortality is due to increased bleeding after envenomation including venom-induced consumption coagulopathy or disseminated intravascular coagulation due to uncontrolled activation of factor V and factor X by pro-coagulants present in the venom (JIACM 2002; 3(4): 392-4).

**Comment**

The citations used by the authors for general description of viper bites cites one basic research article (32) and then the other two describe a European viper and Russell's viper as examples. If Echis is so deadly...the reader would expect literature examples describing Echis. These references are fine, but I was a little surprised. There also seems to be regional variation in the morbidity and mortality from Echis (e.g. fatalities rare in Sri Lanka, even in Jaffna area).Mainly, I think it is important to understand about the NETs, how quickly the inflammatory effects typically manifest as systemic illness and VICC for example. The paper is very strong on the scientific underpinnings, but it is hard to connect this set of dots. I think the story told by these extensive experimental explorations will be enhanced by clinical context. There is no doubt Echis is extremely hard to manage both acutely and chronically. Where does this fit in?

**Response:** As per reviewer suggestions, we have updated references and included in the discussion as follow. There are several case reports suggested that the major complications related to hemotoxic snakebite including *Echis carinatus* (EC). Envenomation by EC induces hematological complications, local pain, bleeding and edema at the bite site. Untreated *Echis* envenomation may involve multiple organs and the patient may suffer from, hematuria, renal failure, hemorrhage, anemia, hypotension and disseminated intravascular coagulation with capillary leak syndrome. The LD50 of Echis carinatus envenomation is 6.65 mg/kg and an average bite may yield about 40 mg of venom (Clinical Toxinology in Asia Pacific and Africa pp 209-232; J. Venom. Anim. Toxins incl. Trop. Dis vol.12 no.1 Botucatu 2006; Am. J. Trop. Med. Hyg., 86(2), 2012, pp. 254–257).

Generally, envenomation by EC is associated with a mortality rate of 10 - 20% if there is no initiation of effective treatment early. The major cause of mortality is due to increased bleeding after envenomation including venom-induced consumption coagulopathy or disseminated intravascular coagulation due to uncontrolled activation of factor V and factor X by pro-coagulants present in the venom (JIACM 2002; 3(4): 392-4).

**Comment**

What are the best case and most likely clinical applications for this intervention?

**Response:** We feel that TTD may be used as small molecule first aid therapy before or along with anti-snake venom administration.

**Reviewer #2**

**Comment**

Are the conclusions supported by the data presented? Generally yes, but see below.

Are the limitations of analysis clearly described? No, and on the basis of the comments indicated below, this will be necessary. The lack of histological assessment of necrosis is a limitation that needs to be discussed. My recommendation, however, is that the authors include this histological analysis to better describe the tissue necrosis.

**Response:** We have included following experimental details, histological analysis to support the manuscript. We have also included the histological assessment data in the form of supplementary information **(Fig. S4)**.

**Fig. S4: Histochemical staining of ECV-induced tissue necrosis in mice footpad and its inhibition by TTD.** Mice footpad was injected with ECV (LD_50_) followed by TTD or DNase 1 injection (30 min post venom injection). Mice were euthanized and footpad tissues were processed for histological sections and analyzed for tissue damage by H & E staining. PBS injected mouse footpad serves as control.

**Comment**

Do the authors discuss how these data can be helpful to advance our understanding of the topic under study? Only partially. It is necessary to better place this study in the context of similar studies assessing the therapeutic potential of SVMP inhibitors.

**Response:** Several scientific reports suggested that, many small inhibitors or chelators of SVMPs such as batimastat, marimastat, N,N,N′,N′-tetrakis (2-pyridylmethyl) ethane-1,2-diamine and Dimercaprol which targets the different classes of SVMPs-induced toxicities (Am. J. Trop. Med. Hyg. 2000;63:313–319; Toxicon. 2004;43:417–424; Biomedicines. 2020 Aug 20;8(9):E297; Toxins (Basel). 2020 May; 12(5): 309; Am. J. Trop. Med. Hyg. 2000;63:313–319; Toxicon. 2004;43:417–424; Commun Biol. 2018 Apr 19;1:34. doi: 10.1038/s42003-018-0039-1. eCollection 2018). Some of these inhibitors were less effective as therapeutic regimen where the time lapse between venom and administration of inhibitors is prolonged (Biochem Pharmacol. 2000 Jul 15;60(2):269-74). Moreover, many small molecules and chelators were reported their interference in Viperinae snake venom-induced coagulopathy and local toxicities (10.3390/biomedicines8090297). However, our findings highlight the efficacy of TTD in ECV-induced both local and systemic toxicities and, would be better repurposing to complement snake bite management.

**Comment**

Is public health relevance addressed? Yes, but it would be relevant to at least mention how would this inhibitor be administered in an eventual clinical use (route of injection).

**Response:** Since TTD is an Antabuse drug and it is given orally, we feel that it will be an oral field treatment as first aid therapy before or along with ASV.

**Comment**

There have been several studies exploring the use of synthetic inhibitors of SVMPs (including both chelating agents and peptidomimetic inhibitors) in experimental models of envenoming by various venoms. The authors need to mention these previous studies, in particular those carried out with Echis sp venoms. When doing this, it is relevant to compare the values of IC50s of some of these inhibitors vs disulfiram, because the concentrations of this inhibitor required to achieve inhibition of venom are in the mM range, while other inhibitors, including chelating agents are in the micromolar range. One wonders why the authors did not test for comparison other SVMP inhibitors previously tested which may be more potent than disulfiram and are commercially available. As mentioned above, it is necessary to find the value of IC50 of disulfiram for proteolytic and hemorrhagic activities through pre-incubation-type experiments.

**Response:** According to the reviewer suggestion we have performed the pre-incubation experiments for inhibition of proteolytic activity of *E. carinatus* venom and hemorrhagic activity. The IC_50_ (median inhibitory concentration) of the TTD for proteolytic activity is 3 mM and IC_50_ for hemorrhagic inhibition is 12 μg and these data included in the form of supplementary figure **(Fig. S3)**.

**Fig. S3: Inhibition of ECV-induced protease activity and local toxicities by TTD.** Reaction mixture (1 ml) contained 0.4 ml of casein (2%) in 0.2 M Tris-HCl buffer pH 8.5 was incubated for 150 min at 37ºC with 25 μg of ECV and various concentrations (0 - 20 mM) of TTD. The inhibition was represented as % inhibition and IC_50_ (median inhibitory concentration) of the TTD was calculated **(A)**. For inhibition of skin hemorrhage, mice were injected (i.d.) with 5 µg of ECV that was pre-incubated with different concentrations of TTD at 37ºC for 5 min. After 180 min, dorsal patches of mice skin were photographed and IC_50_ (median inhibitory concentration) of the TTD was calculated **(B and C)**. For inhibition of tissue necrosis, mice footpads were injected with ECV (LD50; 2.21 mg/kg) pre-incubated with TTD (2.15 mg/kg) at 37ºC for 5 min and footpads were photographed from day 1 to day 8 **(D)**. Red arrow indicates edema and black arrow indicates tissue necrosis. ECV-induced footpad injury was measured manually on a scale of 1 to 5 **(E)**. Data are representative of two independent experiments.

**Comment**

The use of disulfiram has been associated with some adverse effects in patients. This may be mentioned in the Discussion.

**Response:** As per the reviewer suggestions we have included the disulfiram associated some adverse effects in patients in the discussion section of revised manuscript. “TTD/Disulfiram is known to cause hepatitis in 1 in 30,000 patients, which is sometimes fatal. There are rare reports of psychosis and confusional states and peripheral neuropathy and optic neuritis have been reported and, these effects are dose dependent. Moreover, TTD interacts with compounds that utilize the cytochrome P450 enzyme system (Drug Saf. 1999 May;20(5):427-35; Expert Opin Drug Saf. 2008 Jul;7(4):459-72). Although the maximum recommended daily dose of TTD is 500 mg orally as an Antabuse (Meyler's Side Effects of Drugs16th Edition, Editor: Jeffrey K. Aronson, ISBN: 9780444537164), the long-term side effects of its use and dosage requirements are still unknown that requires extensive in vivo research before they can be fully supported as a complementary therapy for snakebite management”.

**Reviewer #3**

**Comment**

(No Response)

Editorial and Data Presentation Modifications?

Use this section for editorial suggestions as well as relatively minor modifications of existing data that would enhance clarity. If the only modifications needed are minor and/or editorial, you may wish to recommend “Minor Revision” or “Accept”.

**Reviewer #1**

(No Response)

**Reviewer #2**

(No Response)

**Reviewer #3**

(No Response)

**Summary and General Comments**

Use this section to provide overall comments, discuss strengths/weaknesses of the study, novelty, significance, general execution and scholarship. You may also include additional comments for the author, including concerns about dual publication, research ethics, or publication ethics. If requesting major revision, please articulate the new experiments that are needed.

**Reviewer #1**

**Comment:** Very nice paper. MR Lewin

**Response:** Authors thank the reviewer.

**Reviewer #2**

**Comment**

This is an interesting contribution that demonstrates that a chelating agent that inhibits SVMPs of Echis carinatus venom inhibits hemorrhagic, necrotizing, and lethal activities even when administered after venom injection, i.e. in rescue-type experiments. This underscores the relevance of SVMPs in the local and systemic effects induced by this venom. These findings are of value. The authors also evaluated the role of SVMPs in the generation of NETosis, which has been shown to be related to the pathogenesis of local necrosis. The possible role of PAR-1 and intracellular pathways in the genesis of NETosis by this venom is also shown. The main concerns I have with this work have been detailed in the comments to specific sections.

**Response:** Authors thank the reviewer.

**Reviewer #3**

**Comment**

Rudresha et al. present a very interesting paper exploring the neutralisation of Echiscarinatus venom (specifically the SVMP toxins) using a repurposed drug (disulfiram). The authors demonstrate interesting efficacy in a variety of animal models (haemorrhage, necrosis and lethality) and also explore the role of NETs in contributing to pathology. Overall, it’s a novel study, and the findings are really quite interesting. There are, however, a number of issues with the paper as it currently stands, though most relate to the text rather than the experimental approach (although see below). Currently, the rationale for various aspects of the work (inhibitor selection, experimental approach, etc) is unclear, with the reader left to piece it together themselves. Important information about the potential clinical utility of the disulfiram vs envenoming is also sadly lacking, including a critical evaluation of its likely utility. Finally, the animal experiments need to be much better defined – I found it impossible to robustly evaluate whether the venom challenges doses used in the lethality experiments were appropriate, for example, while details of the numbers of animals used in each experiment is missing. These are serious problems that currently undermine the manuscript. Despite these issues, there is potential for this study to be a novel and valuable contribution to the field, should the authors address the issues listed below.

**Response:** Authors thank the reviewer.

**Major comments**

**Comment**

The introduction would benefit from some text describing other studies where inhibiting SVMPs has resulted in promising preclinical efficacy vs snakebite envenoming. Reading on, the first 12 lines or so of the results incorporate text that would be useful in the introduction to help the reader understand the rationale better. Please move and integrate this from the results to the introduction. Some rationale for the use of TTD, AA and SLN would be really helpful for the reader – why might these be useful?! It’s not until the first page of the results that I read that TTD has chelating properties, and that AA and SLN are PLA2 and hyaluronidase inhibitors… The rationale for Zn chelation needs to be clearly articulated in the introduction somewhere.

**Response:** As reviewer suggested, we have modified the introduction.

**Comment**

The scoring system used for the footpad experiments requires further detail given the subjective nature of these assessments. Given the nature of these experiments, were any ethical interventions used to reduce pain, suffering and distress of experimental animals? E.g. analgesia? Also, what was the rationale for SCH79797 before venom delivery, while TTD was given post-venom delivery? Why was the venom dose reduced for SCH79797 experiments? This needs to be explained. Finally, what does “LT50” stand for? This is not explained as far as I can see.

**Response:** As per reviewer suggestions, we have given the subjective nature of footpad experiments by providing histological data **(Fig. S4)**. During animal experiments, mice were anesthetized with pentobarbitone (30 mg/kg; i.p.) and then venom/Disulfiram injection was done. However, we have not used any analgesic to treat experimental animals. SCH79797 is a selective antagonist of PAR-1 and it does not have any role in the inhibition of venom-induced toxicities by directly acting on ECV unlike TTD. SVMPs present in ECV can activate PAR-1 in the absence of TTD and once it is activated it is hard to control SVMPs-activated PAR-1 signaling. Hence, we have reduced the venom dose and injected SCH79797 before ECV injection. Moreover, we have not claimed that SCH79797 has therapeutic application in treating ECV-induced toxicities. We made an effort to establish the mechanism of SVMPs in the activation of PAR-1 that may have direct/indirect role in ECV-induced toxicities. We have included this rationale in the results section. According to reviewer 2 suggestions, we have replaced the LT_50_ by LD_50_.

**Comment**

For the mortality studies, the above point about LT50 is important. Why was a known factor of the LD50 (e.g. 5 x LD50) not used? Details of the anti-venom used need to be provided – including source, dose used and route of injection. Finally, for the hemorrhage study: why was the peritoneal cavity only observed in the 2 hour reduced venom challenge experiments, and not the 24 h experiments? Please also change “hemorrhage and bleeding studies” to “hemorrhage study”, and “After 3 h peritoneal” to “after 3 h the animals were euthanized and the peritoneal cavity”

**Response:** According to the reviewer, for the mortality studies “above point about LD_50_” are important, because in LD_50_ venom dosage half of the animals will die. In case administration of “above point about LD_50_” will leads to death of total animals in the group. In our findings, for the mortality studies, we have used 1½LD_50_ dose of *E. carinatus* venom.

**Comment**

All animal experiments need to be clearly defined in terms of numbers of mice used per experimental arm. The details of n numbers also need to be added to the figure legends. There are hints that the haemorrhage experiments are n=2, which is low, but it’s not detailed anywhere, so I cannot assess this.

**Response:** Two independent experiments with 3 animals in each group and this information are included in Methods section and Figure Legends.

**Comment**

I don’t find the degradation gels for laminin (Figure 1, E) or Fibronectin (F) convincing. There are clear lower molecular weight degradation products in the laminin gel even at 20mM TTD concentration, and likewise, for the fibronectin gel the pattern does not match the control. There might be some inhibition, but its not particularly convincing. Also, Panel D in Figure 1 (collagen IV) needs repeating, because there is insufficient clarity in the image due to protein overloading.

**Response**: We do agree with reviewer assessment that degradation gels for laminin and fibronectin was not completely inhibited by TTD. Rather, TTD inhibited degradation of ECV-induced laminin and fibronectin in a dose dependent manner **(Fig. 1E and 1F)**. Moreover, it is very clear that the major bands of laminin and fibronectin were restored in the presence of TTD in concentration dependent manner. According to the reviewer suggestion we have repeated ECV-induced collagen IV degradation and its inhibition by TTD and included as **Fig. 1D** in the revised manuscript.

**Comment**

For the NET formation work, did the authors consider fractionating EC venom to obtain SVMPs to test directly to confirm their hypothesis that SVMPs are likely the causative agents (suggested by the TTD experiments).

**Response:** Fraction of EC venom to test SVMPs action NET formation is of great interest but we feel that it is beyond the scope of this article. Another graduate student is working on the fractionation of EC venom into individual components and test for NETs formation for his PhD work.

**Comment**

In the discussion, the section about TTD requires some additional information. The text about ‘repurposing’ for cancer, AIDS and Lyme disease etc, needs an explanation of the type of studies cited – are the clinical trials? Preclinical studies? Or in vitro studies? Please detail.

**Response:** We have explained the type of studies repurposing TTD for cancer, AIDS and Lyme disease in the discussion as follows, Since then, many studies have shown repurposing of TTD to treat diverse types of human malignant tumors including breast cancer, glioblastoma and pancreatic carcinoma (Curr Pharm Des. 2019;25(30):3248-3256; doi: 10.2174/1381612825666190816233755). TTD has also shown therapeutic potential in treating AIDS and it is found to be beneficial in treating Lyme disease in patients (ClinPharmacolTher. 2019 Mar;105(3):692-702. doi: 10.1002/cpt.1220; Antibiotics (Basel). 2019 May 30;8(2):72. doi: 10.3390/antibiotics8020072).

**Comment**

When discussing other metal chelators you should also cited the paper by Ainsworth et al. 2018 Communications Biology where EDTA was shown to protect against lethality caused by Echis envenoming in vivo.

**Response:** According to the reviewer suggestion, we have included the Ainsworth et al. 2018 Communications Biology in the revised manuscript.

**Comment**

Why did the authors select TTD over other these SVMP inhibitors previously explored (and others, e.g. marimastat, batimastat)? An explanation of the rationale would be helpful. What are the potential drawbacks of TTD in terms of its potential translation for snakebite envenoming? Some discussion of its known PK, tolerance and route of administration should be discussed. What about the doses used in this study? How might they relate to doses required for clinical use? Given that there are a number of safety concerns with the use of TTD – these should be explicitly stated. The discussion would also benefit from the authors stating what needs to be done next to move this promising drug forward.

**Response:** Several scientific reports suggested that, many small inhibitors or chelators of SVMPs such as batimastat, marimastat, N,N,N′,N′-tetrakis (2-pyridylmethyl) ethane-1,2-diamine and Dimercaprol which targets the different classes of SVMPs-induced toxicities (Am. J. Trop. Med. Hyg. 2000;63:313–319; Toxicon. 2004;43:417–424; Biomedicines. 2020 Aug 20;8(9):E297; Toxins (Basel). 2020 May; 12(5): 309; Am. J. Trop. Med. Hyg. 2000;63:313–319; Toxicon. 2004;43:417–424; Commun Biol. 2018 Apr 19;1:34. doi: 10.1038/s42003-018-0039-1. eCollection 2018). Some of these inhibitors were less effective as therapeutic regimen where the time lapse between venom and administration of inhibitors is prolonged (BiochemPharmacol. 2000 Jul 15;60(2):269-74). Moreover, many small molecules and chelators were reported their interference in Viperinae snake venom-induced coagulopathy and local toxicities (10.3390/biomedicines8090297). However, our findings highlight the efficacy of TTD in ECV-induced both local and systemic toxicities and, would be better repurposing to complement snake bite management (Advantage with TTD is that it is a specific zinc chelator).

It has been demonstrated that 80 - 95% of an oral dose of TTD is absorbed from the gastrointestinal tract and it requires 1 to 2 h to peak serum concentration. TTD/Disulfiram is rapidly distributed in tissues including adipose tissue, liver, spleen, adrenal gland and the brain. Prolonged administration of TTD is not known to induce tolerance and it is metabolized to diethyldithiocarbamate and mixed disulfides that are excreted via urine. The unabsorbed content of TTD is excreted in the feces (Journal of Addictions Nursing 21: 54-55, 2010; Expert Opin Drug Saf. 2008 Jul;7(4):459-72).

On the other hand, TTD/Disulfiram is known to cause hepatitis in 1 in 30,000 patients, which is sometimes fatal. There are rare reports of psychosis and confusional states and peripheral neuropathy and optic neuritis have been reported and, these effects are dose dependent. Moreover, TTD interacts with compounds that utilize the cytochrome P450 enzyme system (Drug Saf. 1999 May;20(5):427-35; Expert Opin Drug Saf. 2008 Jul;7(4):459-72).

Although the maximum recommended daily dose of TTD is 500 mg orally as an Antabuse (Meyler's Side Effects of Drugs16th Edition, Editor: Jeffrey K. Aronson, ISBN: 9780444537164), the long-term side effects of its use and dosage requirements are still unknown that requires extensive in vivo research before they can be fully supported as a complementary therapy for snake bite management.

**Minor comments**

**Comment**

Abstract: ECV definition needs explaining.

**Response:** As per reviewer suggestions, we have included the explanation of ECV in revised manuscript.

**Comment**

Introduction: “never the less” should read “nevertheless”

**Response:** As per reviewer suggestions, we have modified the “never the less” to “nevertheless” and included in the revised manuscript.

**Comment**

Introduction: Echis should be italicised

**Response:** According to the reviewer suggestion we have modified the word “Echis” to “*Echis*” in the revised manuscript.

**Comment**

Introduction: “Echis bite is responsible for the highest mortality” – I’m not sure there is any evidence for this in India. If there is, it should be cited. Also, “due to rich in zinc” should read “due to its venom being rich in zinc”

**Response:** We agree with the reviewer suggestion, there are no direct evidences on “*Echis* bite is responsible for the highest mortality in India”. We have replace the sentence “*Echis* bite is responsible for the highest mortality and causes severe local manifestations, due to rich in zinc (Zn^++^)-dependent SVMPs” by “*Echis* bite is responsible for 10 - 20% mortality and causes severe local manifestations, due to rich in zinc (Zn^++^)-dependent SVMPs”(JIACM 2002; 3(4): 392-4) and included in the revised manuscript.

**Comment**

Introduction: “(NETs) the process is described as”, please edit to “(NETs) – this process is described as”

**Response:** According to the reviewer suggestion we have replace the sentence, “(NETs) the process is described as” by“(NETs) this process is described as” and included in the revised manuscript.

**Comment**

Introduction: “they showed that NETs” – who showed this?! Revise to “A previous study showed etc”. Also “lock” should be replaced by “entrap”

**Response:** According to the reviewer suggestion we have revised the “they showed that NETs” by “Katkar et al. showed that NETs” and the word “lock” replaced by “entrap” and included in the revised manuscript.

**Comment**

Introduction: “in this line we have used an Antabuse drug”. This doesn’t follow for the reader – you need to explain why disulfiram was tested based on the findings above. Also, replace “in this line” with “consequently” or something similar.

**Response:** As per the reviewer suggestions we have included the importance of disulfiram in medical consequences in introduction part of the revised manuscript. “Chelating agents are essential in restoring the physiological levels of MMPs, as their dysregulated activity reflects in debilitating conditions such as cancer and arthritis (Cancers (Basel). 2014 Jun 27;6(3):1298-327. doi: 10.3390/cancers6031298; Mol Pharmacol. 2003 Nov;64(5):1076-84. doi: 10.1124/mol.64.5.1076). Many pharmacologically approved chelating agents have been extensively studied for inhibition of SVMPs (Curr Top Med Chem. 2011;11(20):2578-88. doi: 10.2174/156802611797633375; J Endod. 2012 Jan;38(1):62-5. doi: 10.1016/j.joen.2011.09.005). Those molecules are failed to reach the clinical trial, because of their non-specific chelation property (Toxicon. 2013 Sep;71:41-8. doi: 10.1016/j.toxicon.2013.05.013. Epub 2013 May 30). Consequently, a high affinity membrane permeable Zn^++^ chelator, Antabuse drug, disulfiram/TTD repurposed as therapeutic for ECV-induced toxicities in preclinical setup”

**Comment**

Methods: How long were the venoms stored at -20ºC for?

**Response:** We do not store re-suspended venoms for long term. Generally, we store lyophilized venom at -20ºC until further use.

**Comment**

Methods: please detail the sex of the mice used.

**Response:** The animals used in all in-vivo experiments were 6 to 8 week old female Swiss albino mice, (weighing 20 - 25 g).We have mentioned in the method section, “Adult Swiss albino mice (6 to 8 week old female) weighing 20 - 25 g”.

**Comment**

Methods: please add the conditions used for electrophoresis.

**Response:** According to the reviewer suggestion we have explained the conditions used for electrophoresis in the revised manuscript.

**Comment**

Methods: Why were different doses of TTD, AA and SLN given to the mice when assessing haemorrhage? Why were these not consistent to enable comparison?

**Response:** Individual inhibitor is relatively specific to inhibit different enzymatic activity of snake venom. The efficacy of individual inhibitor inhibiting the respective enzymatic activity is different. Hence, we have used different doses of TTD, AA and SLN.

**Comment**

Methods: “analysis of NETs formation and” should read “Analysis of NET formation and”

**Response:** As per the reviewer suggestion we have corrected “analysis of NETs formation and” to “Analysis of NET formation and” and included in the revised manuscript.

**Comment**

Methods: Please detail exactly what the lysis buffer containing EDTA free protease inhibitor mixture contains.

**Response:** We have given the composition of cell lysis buffer and modified this sentence as “The excised tissue samples were sonicated for 30 seconds on ice bath (five passes of 10 seconds) with a 3.0 mm probe sonicator in cold cell lysis buffer containing 20 mM Tris-HCl (pH 7.5), 150 mM NaCl, 1 mM Na_2_EDTA, 1 mM EGTA, 1% Triton, 2.5 mM sodium pyrophosphate, 1 mM beta-glycerophosphate, 1 mM Na_3_VO_4_, 1 µg/ml leupeptin (Cell Signaling Technology) and supplemented with PMSF and phosphatase inhibitor cocktail (Sigma Aldrich).

**Comment**

Methods: For western blotting, please provide the electro-blotting conditions used the concentration of primary antibodies used, and what the TBST buffer contains.

**Response:** As per the reviewer suggestions, we have included the conditions used for the electro-blotting, concentration of primary antibodies in electro-blotting and TBST buffer constituents in the revised manuscript.

**Comment**

Results: Change “TTD also inhibited ECV-induced ECM proteins, collagen, laminin and fibronectin degradation” to “TTD also inhibited ECV-induced degradation of the ECM proteins, collagen, laminin and fibronectin,

**Response:** According to the reviewer suggestion, we have changed the sentence and included in the revised manuscript.

**Comment**

Results: Change “skin hemorrhage in mice skin” to “murine skin hemorrhage”

**Response:** As per the reviewer suggestion we have corrected “skin hemorrhage in mice skin” to “murine skin hemorrhage” phrases of result section and included in the revised manuscript.

**Comment**

Results: Sentence beginning “Recently, Katkar et al. reported” needs rephrasing.

**Response:** As per the reviewer suggestion we have modified the sentence “Recently, Katkar et al. reported” to “A previous study by Katkar et al. reported” and included in the revised manuscript.

**Comment**

Results: “ECV is lethal when injected at 3.31 mg/kg” – please provide the reference supporting this assertion.

**Response:** As per reviewer suggestion, we have given the supporting reference previously published from our laboratory (J Nat Med (2015) 69:111–122). According to our previous study, ECV LD_50_ is 2.21 mg/kg body weight that kills 50% of the animals. In order to induce 100% lethality, we have given 1.5 LD_50_ (3.31 mg/kg) of ECV venom.

**Comment**

Results: “therapeutic mode” might read better as “challenge then treat”

**Response:** As per the reviewer suggestion, we have corrected beginning sentence “therapeutic mode” to “challenge then treat” and included in the revised manuscript.

**Comment**

Results: “this indicates that TTD is a potential drug candidate that complements ASV during EC bite” – reword. No evidence that this is a complementary treatment, instead used as a comparator. Please detail dosage of both TTD and ASV used so that the reader understands this clearly at this point in the manuscript.

**Response:** As per the reviewer suggestions, we have reword the sentence “this indicates that TTD is a potential drug candidate that complements ant-snake venom during EC bite” to “This data indicates that TTD is a potential SVMPs inhibitor that neutralizes the ECV-induced toxicities during EC bite”. According to the reviewer suggestions we have given the details of both TTD and ASV, doses used for this study.

**Comment**

Results: For the neutralization of lethality please describe the data. I.e. both TTD and ASV protected all experimental animals (n=5?) from the lethal effects of the venom in the 24 h co-incubation model. For the challenge then treat model, four of five (?) experimental animals survived when treated with TTD etc.

**Response:** We have described neutralization of lethality data in the results section as “TTD neutralized ECV-induced lethality and protected mice in both pre-incubation (100% survival - two independent experiments with 5 animals in each group) and challenge then treat (30 min post venom injection) (four of five animals survived - two independent experiments with 5 animals in each group) **(Fig. 3A and 3B)**

**Comment**

Results: “*Bothrops bilineata*” should read “*Bothrops bilineatus*”

**Response:** As per the reviewer suggestion “*Bothrops bilineata*” is replaced by “*Bothrops bilineatus*” and included in the revised manuscript.

**Comment**

Results: Sentence beginning “PARs are members” needs editing as currently it doesn’t make sense. Also “relatively MMPs are acted on” also needs editing.

**Response:** According to the reviewer suggestion, we have modified the sentence as “It is well known that MMPs cleave PAR-1 at non-canonical sites, results in the activation of intracellular signaling cascade via MAPKs that leads to a diverse array of physiological functions”.

**Comment**

Figure 3: I suggest that the authors crop the mouse images so that only the exposed peritoneal cavity is visible, and enlarge the images to allow better clarity.

**Response:** As per the reviewer suggestions we have modified the **Fig. 3** (we have crop the mouse peritoneal cavity and enlarged the images to a better clarity) and included in the revised manuscript.

**Comment**

Discussion: “Viper bites predominately cause local tissue necrosis at the bitten site”. Actually this is not the most common sign of pathology – some victims suffer necrosis, but only a small percentage. Rephrase.

**Response:** As per the reviewer suggestion we have rephrased the sentence “Viper bites predominately cause local tissue necrosis at the bitten site” to “Viper bites induce progressive tissue necrosis results in permanent disability at the bitten site” and included in the revised manuscript.

**Comment**

Discussion, “Generally SVMPs contain..” – only the P-III SVMPs contains the D, C and M domains. The rest of the sentence about ADAMDEC-1 is unclear – this isn’t an SVMP. And again, later in the paragraph “the ADAMDEC-1 plays a crucial role in their enzymatic activities”. This is unclear – ADAMDEC-1 is not a domain of SVMPs…

**Response:** As per the reviewers suggestion we have modified the part of discussion accordingly. “SVMP contains disintegrin-like (D), cysteine-rich (C), metalloproteinase and (M) domain, that harbors putative Zn^++^ binding sequence and bearing the typical structural features of the metzincin family of MMPs. Alike MMPs, Zn^++^ on the M-domain of SVMPs plays a crucial role in the catalytic functions. Hence, employing specific Zn^++^ chelating agents will be a better approach to counteract the debilitating action of SVMPs. In addition, chelation of Zn^++^ metal ion from M-domain of SVMPs, by specific Zn^++^chelators rather than non-specific chelator is more effective in the management of local tissue necrosis induced by viper venoms”

**Comment**

Discussion: “chelation of Zn metal ion from SVMPs by specific Zn chelators rather than anti-venom like molecule is more effective in the management…” Is it? What is the evidence for this?

**Response:** There are no direct evidences to support the sentence “chelation of Zn^++^ metal ion from SVMPs, by specific Zn^++^ chelators rather than anti-venom like molecule is more effective in the management.” We have rephrased this sentence to “chelation of Zn^++^ metal ion from SVMPs, by specific Zn^++^ chelators rather than non-specific chelator is more effective in the management” ….(Toxicon. 2015 Jan;93:68-78. doi: 10.1016/j.toxicon.2014.11.224. Epub 2014 Nov 8).
