# Supplementary material for: Echis carinatus snake venom metalloprotease-induced toxicities in mice: Therapeutic intervention by a repurposed drug, Tetraethyl thiuram disulfide (Disulfiram)
Source: PLoS Negl Trop Dis. 2021 Feb 2;15(2):e0008596. doi: 10.1371/journal.pntd.0008596 (PMC7880489; doi:10.1371/journal.pntd.0008596)
Supplement: Attachment — Submitted filename: PNTD R2 response to reviewer.docx [file pntd.0008596.s008.docx]

The authors would like to thank the editor for considering our manuscript for revision in the journal PLOS Neglected Tropical Diseases. We also thank all the reviewers for critically evaluating the manuscript. The comments raised by reviewers have been addressed as detailed below.

**Comment**

The reason for this is that both myself and Deputy editor are concerned regarding the ethics of the footpad model employed in this study. Moreover, all the reviewers, at one moment or another, have additionally questioned the ethical problem of this model. It is evident from the photographs in the figures that the mice would have experienced substantial pain and distress for an extended period.

From the results presented in Fig 2 A/B/C, Fig 6 and Fig S3 D/E, it is difficult to understand the requirement to expand the experiment past day 4. Is the data obtained by day 4 not sufficient to demonstrate a clear conclusion? It is sufficient, then it is unnecessary to extend the suffering of the animals to day 8.

We are therefore requesting further addition of discussion and clarification regarding this model.

Specifically:

1) you need to unequivocally demonstrate, using strong arguments, that it is essential to prolong the monitoring of the mice in the footpad model for 8 days.

Or

2) Demonstrate that you recognize that the elements obtained on the fourth day (or earlier) are sufficient to obtain a clear conclusion without difference to that of the eighth day, and therefore that it becomes unnecessary to prolong the suffering of the mice beyond this point.

We recognize the urgent need for research investigating anti-necrotic snakebite therapies, and that due to the complex pathology of envenoming animal models are currently a necessity. However, it is essential to find a way to avoid the suffering of the mice during the entire duration of such experiments and not just at the time of the injection of the venom. If analgesia was used throughout the experiment, this has not been stated yet. Considerations of refining the protocol for future use (by yourselves or others) need to be included.

**Response:** The authors would like to assure that all the experimental protocol related to animals was discussed before the Institutional Animal Ethical Committee approved by the regulatory body “Committee for the Purpose of Control and Supervision of Experiments on Animals (CPCSEA)”. The regulatory body CPCSEA is approved as an Act of Parliament, by Government of India. Once again, we reiterate that the experiments were carried out to benefit the mankind and the animal world at large. Based on these results an antiserum company in India are conducting a trial to treat the wounds developed in horses during anti-venom production.

The inevitable problem of local toxicity by *E. carinatus* venom is due to host physiological self defence process of NETosis as described by Prof. K. Kemparaju and his team from our Department. To address this problem, Prof. K. Kemparaju and his team devised an animal (mice) model by injecting *E. carinatus* venom into the tail region to observe the time kinetics of progressive necrosis. In this experiment they observed the detachment of necrotized tail between 8 - 10 days time.

In this line, we have designed similar two animal studies in mouse and rabbit.

1. In mouse model, *E. carinatus* venom was injected into the footpad to follow the time kinetics of progressive necrosis. Due to necrosis the loss of toes / fingers in the mouse foot pad was observed between 6 - 8 days.

2. In rabbit model, *E. carinatus* venom was injected into the rabbit skin since the rabbit skin closely resembles that of human and followed the time kinetics of progressive necrosis. The tissue necrosis was observed up to 14 days. These experimental protocols of mouse and rabbit were part of publication in the Journal Toxicon (Toxicon. 2020 Aug;183:1-10) of our earlier study. To be consistent with our published protocols the experiments was carried out for eight days.

Since the mouse model is already published and footpad necrosis was followed up to 8 days, if we conclude the results at the end of 4 days there will always a possibility of comments from the reviewer for not following up to 8 days quoting our published data. In such scenario repeating the experiments involves more number of animals. The study conducted conclusively neutralizes the necrotizing toxicity caused by *E. carinatus* venom upon administration.

During this experiment no analgesic drugs were used. It is difficult to rule out the possible influence of analgesic drugs in neutralizing the venom induced local toxicity by TTD.

**Methods**

-Are the objectives of the study clearly articulated with a clear testable hypothesis stated?

-Is the study design appropriate to address the stated objectives?

-Is the population clearly described and appropriate for the hypothesis being tested?

-Is the sample size sufficient to ensure adequate power to address the hypothesis being tested?

-Were correct statistical analysis used to support conclusions?

-Are there concerns about ethical or regulatory requirements being met?

**Reviewer #1**

**Comment**

This is both impressive and done with sufficient detail that I feel as if I could readily reproduce or extend the studies described. I appreciate the diligent methodology and reporting.

**Response:** Authors thank the reviewer.

**Reviewer #2**

**Comment**

The authors have adequately addressed my main concerns with the methods and have modified the paper accordingly. The inclusion of experiments with preincubation of venom and inhibitor was done.

**Response:** Authors thank the reviewer.

**Reviewer #3**

(No Response)

**Results**

-Does the analysis presented match the analysis plan?

-Are the results clearly and completely presented?

-Are the figures (Tables, Images) of sufficient quality for clarity?

**Reviewer #1**

Yes. See below.

**Reviewer #2**

**Comment**

No major comments, except that I suggest the authors express the inhibitory activity on proteolysis and haemorrhage of the chelating agent in molar terms, i.e. expressing the Median Inhibitory Concentration (IC50) in molar terms. In the manuscript they present a graph but there is no reference to IC50. In order to compare this inhibitor with others, and to better express its inhibitory activity, IC50 should be expressed in molar terms in experiments where venom and inhibitors were preincubated. The authors already have the information to do so.

**Response:** According to the reviewer suggestion we have expressed the inhibitory activity on haemorrhage of TTD (chelating agent) in molar terms (i.e., IC50 = 5.7 mM).

And also, as per the reviewer suggestion we have expressed the inhibitor concentration of inhibitor (TTD, AA and SLN) in molar terms in experiments where venom and inhibitor were pre-incubated and included in the revised manuscript.

**Reviewer #3**

(No Response)

**Conclusions**

-Are the conclusions supported by the data presented?

-Are the limitations of analysis clearly described?

-Do the authors discuss how these data can be helpful to advance our understanding of the topic under study?

-Is public health relevance addressed?

**Reviewer #1**

**Comment**

Yes. See below.

**Reviewer #2**

**Comment**

The conclusions are sound and supported by the data.

**Response:** Authors thank the reviewer.

**Reviewer #3**

(No Response)

**Editorial and Data Presentation Modifications?**

Use this section for editorial suggestions as well as relatively minor modifications of existing data that would enhance clarity. If the only modifications needed are minor and/or editorial, you may wish to recommend “Minor Revision” or “Accept”.

**Reviewer #1**

(No Response)

**Reviewer #2**

(No Response)

**Reviewer #3**

(No Response)

**Summary and General Comments**

Use this section to provide overall comments, discuss strengths/weaknesses of the study, novelty, significance, general execution and scholarship. You may also include additional comments for the author, including concerns about dual publication, research ethics, or publication ethics. If requesting major revision, please articulate the new experiments that are needed.

**Reviewer #1**

**Comment**

This is both impressive and done with sufficient detail that I feel as if I could readily reproduce or extend the studies described. I appreciate the diligent methodology and reporting.

I make two comments

Discussion: The LD50s (refs 50-52) do not match well with what you would expect if the effect of the venom was linear e.g. 6.5mg/kg and venom yield of 40mg... would kill 1/2 the people weighing six or seven Kg. What I think the authors intend from their work and other elements of the paper is to show that the effect of a small amount of venom produces an outsized effect on human victims through cascades of provoked inflammation. What is needed to produce lethality in mice is not necessary to make this argument coherent...mice and humans are not comparable for purposes of susceptibility to inflammation or the response to venom. Noting this does not in any way diminish the methods or overall conclusions. I would say it strengthens the arguments that pharmacological interventions that can neutralize venom and/or simply blunt the victims dysregulated response to venom or venom-induced tissue injury helps the case. Based on the reported LD50 (at least as stated), you would need much much bigger snakes to have serious lethality. A 40mg dose of venom is a relative under-dose. What is remarkable is how big an injury and degree of lethality that little amount of venom can produce.

The vast majority of snakebites are from defensive strikes and minimal venom is injected. Yet, despite this massive "under-dosing" the bites produce devastating systemic effects (likely because of dysregulated host response--humans are not the intended victims of venom). Saw scale vipers are a particularly notable case. The patients rarely die quickly. The major damage occurs days or weeks after the bite. Understanding this is key to understanding the relevance of the work and for the authors to show why understanding the mechanisms--particularly for saw scale--is critical. A more explicit statement of the natural history of the bites would be helpful to put your findings in context for general readers.

**Response:** Generally, in order to see the effect of any molecule the experiments are standardized to attain suboptimal conditions like IC_50_ in this study LD_50_. For studies like hemorrhagic activity there is protocol to determine minimum hemorrhagic dose (MHD). For edema study no such protocols are available. We relied on LD_50_ dose to begin with to determine the effect of TTD in neutralizing local inflammation. We agree with the author that toxicity study is definitely not linear. The LD_50_ dose for *E. carinatus* venom is 2.21 mg/kg. Theoretically, one can extrapolate LD_25_, LD_75_ and LD_100_. But in reality the venom concentration little above LD_50_ would kill all the animals in that group and similarly the venom concentration just below LD_50_, all animals will survive in that group.

The symptoms and toxicity observed in mice are extrapolated to humans since the clinical studies on snake venom envenomation in humans is not studied well. Moreover, as reviewer stated that saw scale vipers bite rarely leads to the death of the victim quickly. And also, the major damage including local tissue necrosis occurs days or weeks after the bite.

As we mentioned earlier an antiserum company in India are conducting a trial to treat the wounds developed by few horses during anti-serum production our fingers are crossed and hoping for positive results.

**Comment**

I think this reference is an omission and should be included (I am not any of these people): It probably belongs in the bibliography around reference 54 or so.

Brust A, Sunagar K, Undheim EA, Vetter I, Yang DC, Casewell NR, Jackson TN, Koludarov I, Alewood PF, Hodgson WC, Lewis RJ, King GF, Antunes A, Hendrikx I, Fry BG. Differential evolution and neofunctionalization of snake venom metalloprotease domains. Mol Cell Proteomics. 2013 Mar;12(3):651-63.

**Response:** As per the reviewer suggestion we have included the suggested reference (Mol Cell Proteomics. 2013 Mar;12(3):651-63. doi: 10.1074/mcp.M112.023135. Epub 2012 Dec 12) in the revised manuscript.

**Comment**

Superb team effort to put this all together.

Matthew R. Lewin, MD, PhD

California Academy of Sciences

**Response:** Authors thank the reviewer.

**Reviewer #2**

(No Response)

**Reviewer #3**

**Comment**

It’s nice to see that the authors have really engaged with the various reviewer suggestions and have integrated a number of changes into the manuscript. I am happy for the manuscript to be accepted, pending some minor corrections outlined below. The manuscript would also benefit from some English language improvements, which hopefully PLOS NTD will be able to facilitate. I congratulate the authors on a very interesting piece of work.

**Response:** We have improved English language of the revised manuscript.

**Suggested minor changes**

**Comment**

Author summary: “Here we investigated the ECVMPs are responsible for”. ECVMP has not been defined at this point.

**Response:** As per the reviewer suggestion, we have defined the ECVMPs in the author summary and included in the revised manuscript.

**Comment**

Methods, in vivo experiments and elsewhere: “challenging” studies, would be better as “challenge studies”

**Response:** As per reviewer suggestion, we have modified the “challenging studies” to “challenge studies” in method section of the revised manuscript.

**Comment**

Discussion, first line: Would read better as: “Viper bites can induce progressive tissue necrosis that can result in permanent disability in the affected limb or digit”

**Response:** As per the reviewer suggestion we have modified the “Viper bites induce progressive tissue necrosis results in permanent disability at the bitten site” to “Viper bites can induce progressive tissue necrosis that can result in permanent disability in the affected limb or digit” and included in the revised manuscript.

**Comment**

Discussion, second line: this sentence (beginning “there are several case reports”) needs rephrasing as it doesn’t currently make sense.

**Response:** As per the reviewer suggestion we have modified the sentence “There are several case reports suggested that the major complications related to hemotoxic snakebite including Echis carinatus (EC)” to “Case reports on snake bite victims suggested that envenomation by hemotoxic venoms including Echis carinatus induces haematological complications, local pain, bleeding and edema at the bite site”.

**Comment**

Discussion, lines 3-6: citations are needed for this information about Echis carinatus envenoming.

**Response:** According to the reviewer suggestion we have include the suitable reference in the discussion section (Lines 3-6) (Semin Nephrol. 2008 Jul;28(4):363-72; J Assoc Physicians India. 2004 Jan;52:63-4)

**Comment**

Discussion, line 7: given the text about human envenoming above, the LD50 information needs to be clearer that this relates to animal studies! i.e. “in mice”

**Response:** The reported LD50 of EC envenomation (i.e., 6.65 mg/kg) is based on the published reports.

**Comment**

Discussion, line 11-12: my understanding is that Echis envenoming predominately act in a procoagulant manner by activating prothrombin, not Factor V or X.

**Response:** As per the reviewer suggestion we have modified the “The major cause of mortality is due to increased bleeding after envenomation including venom-induced consumption coagulopathy or disseminated intravascular coagulation due to uncontrolled activation of factor V and factor X by pro-coagulants present in the venom” to “The major cause of mortality is due to increased bleeding after envenomation including venom-induced consumption coagulopathy or disseminated intravascular coagulation due to the prothrombin/thrombin-like enzymes present in the snake venom” and included in the revised manuscript.

**Comment**

My final suggestion would be that the authors write a final paragraph summarising the wider implications of their interesting findings and what should be done next to progress their work. Currently, the paper finishes quite abruptly.

**Response:** As per the reviewer suggestion we have included the final paragraph in the form of conclusion in the revised manuscript.

Conclusion

There is an urgent need for effective snakebite treatments that can be administered in the remote areas where medical access is limited and also that can complement ASV. The current findings suggested that TTD is a pharmacologically approved an Antabuse drug and that inhibits ECVMPs-induced NETosis in human neutrophils and footpad tissue necrosis in mice. Moreover, TTD also neutralized ECV-induced systemic hemorrhage and conferred protection against lethality in mice. Furthermore, we demonstrated that ECVMPs-induced NETosis and tissue necrosis is mediated via PAR-1-ERK axis. Overall, our results provide an insight into SVMPs-induced toxicities and the promising neutralizing potency of TTD can be exploited as first aid therapy, complementing ASV to treat snakebite-induced toxicities.

**Comment**

Accompanying raw data underpinning the manuscript should be made available in some form.

**Response:** As per the reviewer suggestion we have included raw data related to electrophoretic gels and Western blots at the end of the response to the reviewer.

**Do you want your identity to be public for this peer review?**

**Reviewer #1**

Yes: Matthew R Lewin

Authors thank the reviewer.

**Reviewer #2**

No

**Reviewer #3**

No

**Raw data**
